# Supplementary material for: Modeling glioblastoma heterogeneity as a dynamic network of cell states
Source: Mol Syst Biol. 2021 Sep 16;17(9):e10105. doi: 10.15252/msb.202010105 (PMC8444284; doi:10.15252/msb.202010105)
Supplement: Supplementary file 5 — Source Data for Figure 3 [file MSB-17-e10105-s001.zip › Figure3A_sourcedata/GSEA_3065/hallmarks_state6.GseaPreranked.1623416308702/HALLMARK_UNFOLDED_PROTEIN_RESPONSE.html]

Details for gene set HALLMARK\_UNFOLDED\_PROTEIN\_RESPONSE[GSEA]

|  || Dataset | state6 |
| Phenotype | NoPhenotypeAvailable |
| Upregulated in class | na\_neg |
| GeneSet | HALLMARK\_UNFOLDED\_PROTEIN\_RESPONSE |
| Enrichment Score (ES) | -0.36410758 |
| Normalized Enrichment Score (NES) | -1.4433774 |
| Nominal p-value | 0.009404388 |
| FDR q-value | 0.062269308 |
| FWER p-Value | 0.541 |
Table: GSEA Results Summary

  

Fig 1: Enrichment plot: HALLMARK\_UNFOLDED\_PROTEIN\_RESPONSE      
 Profile of the Running ES Score & Positions of GeneSet Members on the Rank Ordered List

  

| PROBE | GENE SYMBOL | GENE\_TITLE | RANK IN GENE LIST | RANK METRIC SCORE | RUNNING ES | CORE ENRICHMENT || 1 | EEF2 |  |  | 20 | 0.286 | 0.0318 | No |
| 2 | RPS14 |  |  | 72 | 0.229 | 0.0532 | No |
| 3 | YWHAZ |  |  | 292 | 0.153 | 0.0462 | No |
| 4 | DDIT4 |  |  | 357 | 0.142 | 0.0558 | No |
| 5 | ATF3 |  |  | 494 | 0.124 | 0.0549 | No |
| 6 | NHP2 |  |  | 598 | 0.114 | 0.0567 | No |
| 7 | CEBPG |  |  | 640 | 0.111 | 0.0652 | No |
| 8 | CXXC1 |  |  | 645 | 0.111 | 0.0779 | No |
| 9 | IMP3 |  |  | 683 | 0.107 | 0.0864 | No |
| 10 | LSM4 |  |  | 728 | 0.103 | 0.0936 | No |
| 11 | SLC1A4 |  |  | 741 | 0.103 | 0.1045 | No |
| 12 | ATF4 |  |  | 777 | 0.100 | 0.1124 | No |
| 13 | DCP2 |  |  | 830 | 0.096 | 0.1179 | No |
| 14 | ASNS |  |  | 850 | 0.094 | 0.1269 | No |
| 15 | PAIP1 |  |  | 900 | 0.091 | 0.1321 | No |
| 16 | GOSR2 |  |  | 910 | 0.090 | 0.1418 | No |
| 17 | MTHFD2 |  |  | 926 | 0.090 | 0.1508 | No |
| 18 | CEBPB |  |  | 943 | 0.089 | 0.1596 | No |
| 19 | KIF5B |  |  | 1112 | 0.080 | 0.1498 | No |
| 20 | CNOT6 |  |  | 1126 | 0.079 | 0.1577 | No |
| 21 | NOLC1 |  |  | 1181 | 0.076 | 0.1606 | No |
| 22 | LSM1 |  |  | 1425 | 0.067 | 0.1406 | No |
| 23 | ATP6V0D1 |  |  | 1532 | 0.063 | 0.1359 | No |
| 24 | SERP1 |  |  | 1912 | 0.050 | 0.0983 | No |
| 25 | EXOSC1 |  |  | 1936 | 0.050 | 0.1016 | No |
| 26 | EIF4G1 |  |  | 2046 | 0.046 | 0.0946 | No |
| 27 | DKC1 |  |  | 2283 | 0.039 | 0.0721 | No |
| 28 | PSAT1 |  |  | 2600 | 0.031 | 0.0394 | No |
| 29 | EIF4EBP1 |  |  | 2837 | 0.026 | 0.0153 | No |
| 30 | KHSRP |  |  | 2853 | 0.025 | 0.0166 | No |
| 31 | DCTN1 |  |  | 2857 | 0.025 | 0.0193 | No |
| 32 | EIF4A2 |  |  | 2864 | 0.025 | 0.0215 | No |
| 33 | EXOSC4 |  |  | 2877 | 0.025 | 0.0231 | No |
| 34 | MTREX |  |  | 2891 | 0.024 | 0.0245 | No |
| 35 | ALDH18A1 |  |  | 2925 | 0.024 | 0.0235 | No |
| 36 | XPOT |  |  | 2982 | 0.022 | 0.0197 | No |
| 37 | CKS1B |  |  | 2985 | 0.022 | 0.0221 | No |
| 38 | PREB |  |  | 3212 | 0.017 | -0.0018 | No |
| 39 | SHC1 |  |  | 3621 | 0.009 | -0.0476 | No |
| 40 | EXOSC2 |  |  | 4037 | 0.000 | -0.0953 | No |
| 41 | SDAD1 |  |  | 4213 | -0.003 | -0.1150 | No |
| 42 | DCP1A |  |  | 4228 | -0.004 | -0.1162 | No |
| 43 | GEMIN4 |  |  | 4232 | -0.004 | -0.1161 | No |
| 44 | WIPI1 |  |  | 4237 | -0.004 | -0.1161 | No |
| 45 | XBP1 |  |  | 4331 | -0.006 | -0.1261 | No |
| 46 | EIF4A3 |  |  | 4420 | -0.007 | -0.1354 | No |
| 47 | EXOSC5 |  |  | 4644 | -0.012 | -0.1596 | No |
| 48 | ARFGAP1 |  |  | 4654 | -0.012 | -0.1593 | No |
| 49 | ZBTB17 |  |  | 4696 | -0.013 | -0.1625 | No |
| 50 | PARN |  |  | 4720 | -0.013 | -0.1636 | No |
| 51 | NOP14 |  |  | 4771 | -0.014 | -0.1677 | No |
| 52 | SLC30A5 |  |  | 4783 | -0.014 | -0.1673 | No |
| 53 | DDX10 |  |  | 4909 | -0.017 | -0.1797 | No |
| 54 | SLC7A5 |  |  | 4959 | -0.017 | -0.1832 | No |
| 55 | EXOSC10 |  |  | 4960 | -0.017 | -0.1812 | No |
| 56 | EIF4E |  |  | 5088 | -0.020 | -0.1934 | No |
| 57 | CNOT2 |  |  | 5208 | -0.022 | -0.2044 | No |
| 58 | RRP9 |  |  | 5380 | -0.026 | -0.2210 | No |
| 59 | NPM1 |  |  | 5412 | -0.027 | -0.2214 | No |
| 60 | STC2 |  |  | 5456 | -0.028 | -0.2230 | No |
| 61 | SEC31A |  |  | 5520 | -0.029 | -0.2268 | No |
| 62 | VEGFA |  |  | 5660 | -0.032 | -0.2390 | No |
| 63 | ERO1A |  |  | 5668 | -0.032 | -0.2360 | No |
| 64 | EIF2S1 |  |  | 5897 | -0.036 | -0.2579 | No |
| 65 | DNAJB9 |  |  | 5974 | -0.037 | -0.2622 | No |
| 66 | HYOU1 |  |  | 6028 | -0.039 | -0.2637 | No |
| 67 | TTC37 |  |  | 6163 | -0.042 | -0.2741 | No |
| 68 | EXOC2 |  |  | 6494 | -0.050 | -0.3061 | No |
| 69 | NFYB |  |  | 6609 | -0.053 | -0.3129 | No |
| 70 | TATDN2 |  |  | 6681 | -0.055 | -0.3146 | No |
| 71 | HERPUD1 |  |  | 6698 | -0.056 | -0.3098 | No |
| 72 | EXOSC9 |  |  | 6932 | -0.063 | -0.3291 | No |
| 73 | TUBB2A |  |  | 7078 | -0.067 | -0.3378 | No |
| 74 | SRPRB |  |  | 7090 | -0.067 | -0.3310 | No |
| 75 | YIF1A |  |  | 7353 | -0.076 | -0.3520 | No |
| 76 | EIF4A1 |  |  | 7459 | -0.080 | -0.3546 | Yes |
| 77 | BAG3 |  |  | 7481 | -0.080 | -0.3474 | Yes |
| 78 | SSR1 |  |  | 7569 | -0.085 | -0.3474 | Yes |
| 79 | WFS1 |  |  | 7649 | -0.088 | -0.3459 | Yes |
| 80 | BANF1 |  |  | 7787 | -0.095 | -0.3504 | Yes |
| 81 | EDEM1 |  |  | 7798 | -0.096 | -0.3402 | Yes |
| 82 | SRPRA |  |  | 7981 | -0.106 | -0.3485 | Yes |
| 83 | POP4 |  |  | 8090 | -0.113 | -0.3475 | Yes |
| 84 | HSPA9 |  |  | 8097 | -0.113 | -0.3347 | Yes |
| 85 | ATF6 |  |  | 8167 | -0.119 | -0.3285 | Yes |
| 86 | KDELR3 |  |  | 8317 | -0.134 | -0.3296 | Yes |
| 87 | CNOT4 |  |  | 8325 | -0.135 | -0.3144 | Yes |
| 88 | DNAJC3 |  |  | 8407 | -0.147 | -0.3061 | Yes |
| 89 | SEC11A |  |  | 8542 | -0.171 | -0.3011 | Yes |
| 90 | NOP56 |  |  | 8619 | -0.199 | -0.2862 | Yes |
| 91 | SPCS3 |  |  | 8633 | -0.204 | -0.2634 | Yes |
| 92 | SPCS1 |  |  | 8668 | -0.222 | -0.2409 | Yes |
| 93 | FKBP14 |  |  | 8700 | -0.238 | -0.2161 | Yes |
| 94 | FUS |  |  | 8731 | -0.268 | -0.1877 | Yes |
| 95 | CALR |  |  | 8773 | -0.330 | -0.1530 | Yes |
| 96 | HSPA5 |  |  | 8785 | -0.369 | -0.1103 | Yes |
| 97 | PDIA6 |  |  | 8792 | -0.448 | -0.0576 | Yes |
| 98 | HSP90B1 |  |  | 8794 | -0.487 | 0.0002 | Yes |
Table: GSEA details [plain text format]

  

Fig 2: HALLMARK\_UNFOLDED\_PROTEIN\_RESPONSE: Random ES distribution      
 Gene set null distribution of ES for **HALLMARK\_UNFOLDED\_PROTEIN\_RESPONSE**
